# Atrial Myxoma in a Patient With Chronic Obstructive Pulmonary Disease (COPD): Unmasking Overlapping Symptomatology

**DOI:** 10.7759/cureus.55974

**Published:** 2024-03-11

**Authors:** Anas Mahmoud, Mawada Tarhuni, Tala Beilani, Ibrahim Ismail-Sayed, Michael Pelidis

**Affiliations:** 1 Internal Medicine, St. Joseph’s University Medical Center, Paterson, USA; 2 Internal Medicine, California Institute of Behavioral Neurosciences & Psychology, Fairfield, USA; 3 Oncology, Kansas City University, Kansas City, USA; 4 Internal Medicine, St. Luke's University Health Network, Bethlehem, USA; 5 Internal Medicine, St. Joseph's University Medical Center, Paterson, USA

**Keywords:** atrial myxoma, chronic obstructive pulmonary disease(copd), copd, dyspnea on exertion, exertional dyspnea

## Abstract

Atrial myxoma, though the most common primary cardiac tumor, often presents with nonspecific symptoms that can obscure its diagnosis. This case report details an unusual presentation of dyspnea on exertion (DOE) in a patient initially considered to have chronic obstructive pulmonary disease (COPD), a common pulmonary etiology of DOE. The diagnostic journey underscores the critical importance of considering atrial myxoma in patients with DOE, especially when symptoms are not fully explained by apparent pulmonary conditions. Our findings highlight the necessity of a comprehensive diagnostic approach, including the early use of resting transthoracic echocardiogram, to unveil less common causes like atrial myxoma. This case reinforces the pivotal role of considering alternative diagnoses in complex presentations of DOE, thereby guiding more accurate and tailored patient management.

## Introduction

Chronic obstructive pulmonary disease (COPD) is a prevalent and easily identifiable cause of both acute and chronic dyspnea on exertion (DOE) [1]. Despite its commonality, COPD can obscure the diagnosis of other, less frequent etiologies, presenting a significant clinical challenge. In this context, we present a compelling case characterized by intermittent DOE, underscoring the importance of a thorough diagnostic evaluation to uncover hidden conditions.

DOE, defined as the sensation of breathlessness during activities like talking, walking, or performing daily tasks, often stems from either pulmonary or cardiac origins [2]. Among pulmonary causes, conditions such as COPD, characterized by progressive airway obstruction, and asthma, marked by recurrent bronchoconstriction, are noteworthy contributors [3]. Additionally, interstitial lung diseases, pulmonary hypertension, and various disorders affecting lung tissue further exemplify the diverse pulmonary etiologies leading to exertional dyspnea [2,3].

On the cardiac front, especially in patients with established pulmonary disease, distinguishing DOE's root cause becomes more intricate [4]. Valvular issues, a relatively common cardiac origin for DOE, become particularly apparent when classical symptoms align with a clear clinical picture [4]. Notably, atrial myxoma, the most prevalent primary cardiac tumor [5], exhibits variable presentations, ranging from asymptomatic cases to heart failure symptoms such as DOE, orthopnea, or fluid overload symptoms [5,6,7].

Our case highlights that although rare, DOE in a patient with a common pulmonary can have an underlying cause. Furthermore, it underscores the importance of a comprehensive initial workup including a resting transthoracic echocardiogram to rule out potential alternative causes of dyspnea.

In the subsequent sections, we delve into the nuances of these pulmonary and cardiac causes, emphasizing the necessity for thorough evaluations and age-appropriate screening imaging to enhance diagnostic precision and guide tailored management strategies.

## Case presentation

**Figure 1:**
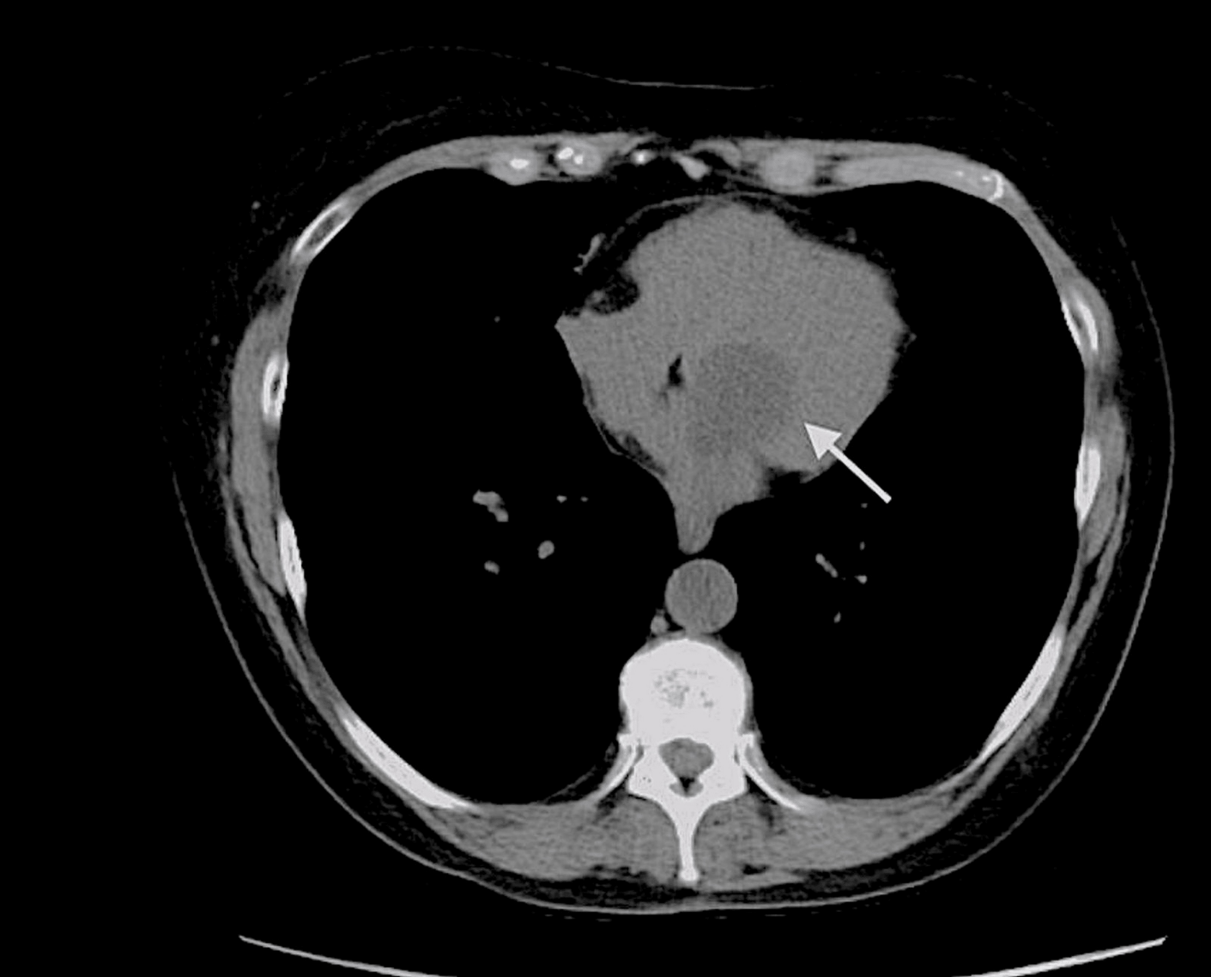
CT of mass near the left atrium

A 59-year-old female with a history of COPD and 40 packs/year of smoking tobacco cigarettes presented to the pulmonary clinic to establish care. She reported occasional mild dyspnea and mild dizziness only with high-intensity exercise, chronic moderate productive cough with yellowish sputum in the morning, while denied any, weight loss, hemoptysis, or family history of cancer. Previous pulmonary function testing (PFT) was consistent with mild COPD (FEV1 of 71%). The physical exam was significant for mild scattered wheezing. She was recommended to continue using inhaled corticosteroids (ICS) combined with the long-acting beta-agonists (LABA) inhaler and repeat PFT. Computed tomography (CT) for lung cancer screening was done; yielding an incidental finding of atrial myxoma. An incidental hypodense 4 x 3.3 cm lesion was noted near the left atrium (Figure 1).

**Figure 2:**
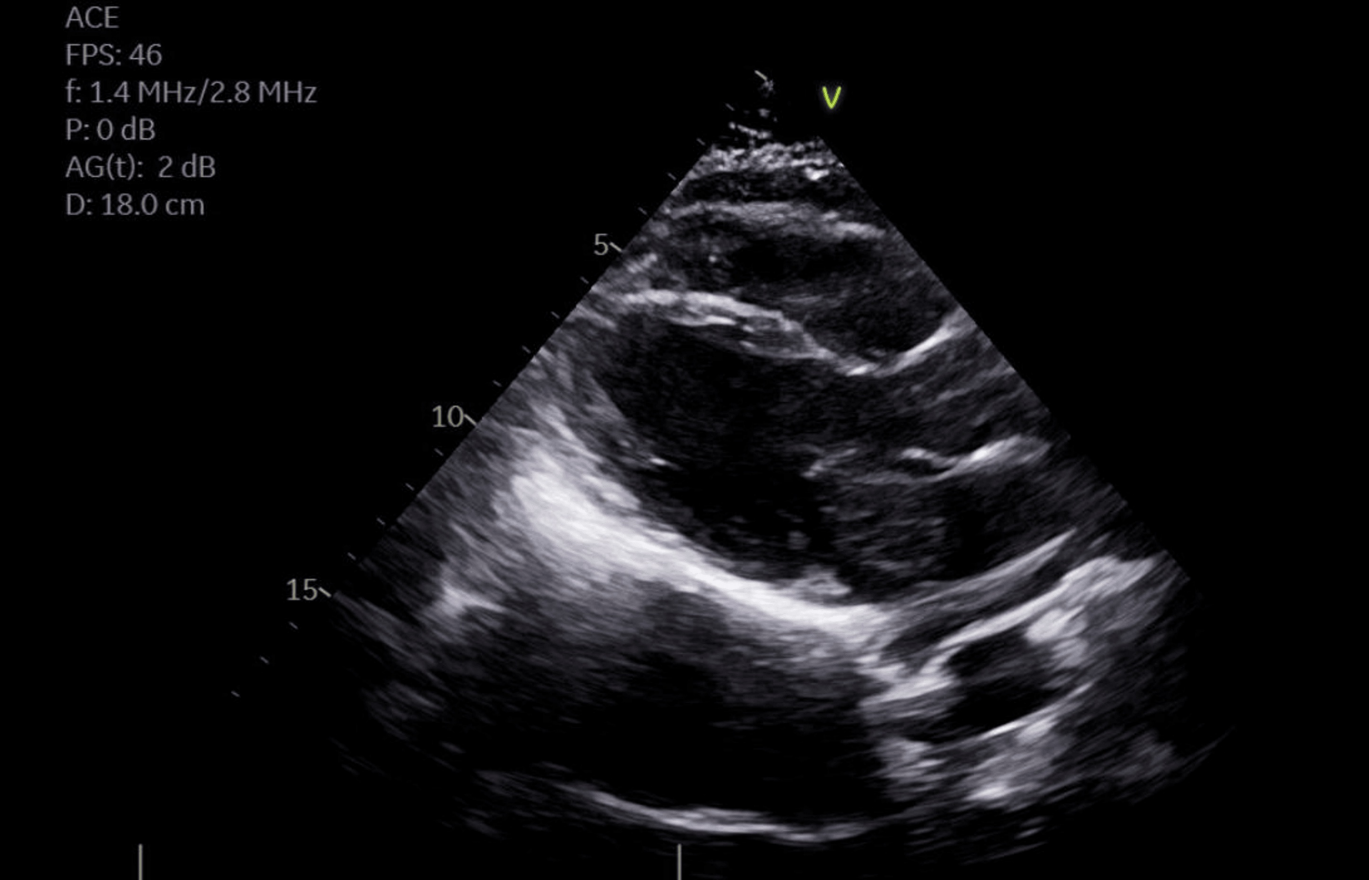
The transthoracic echocardiogram

**Figure 3:**
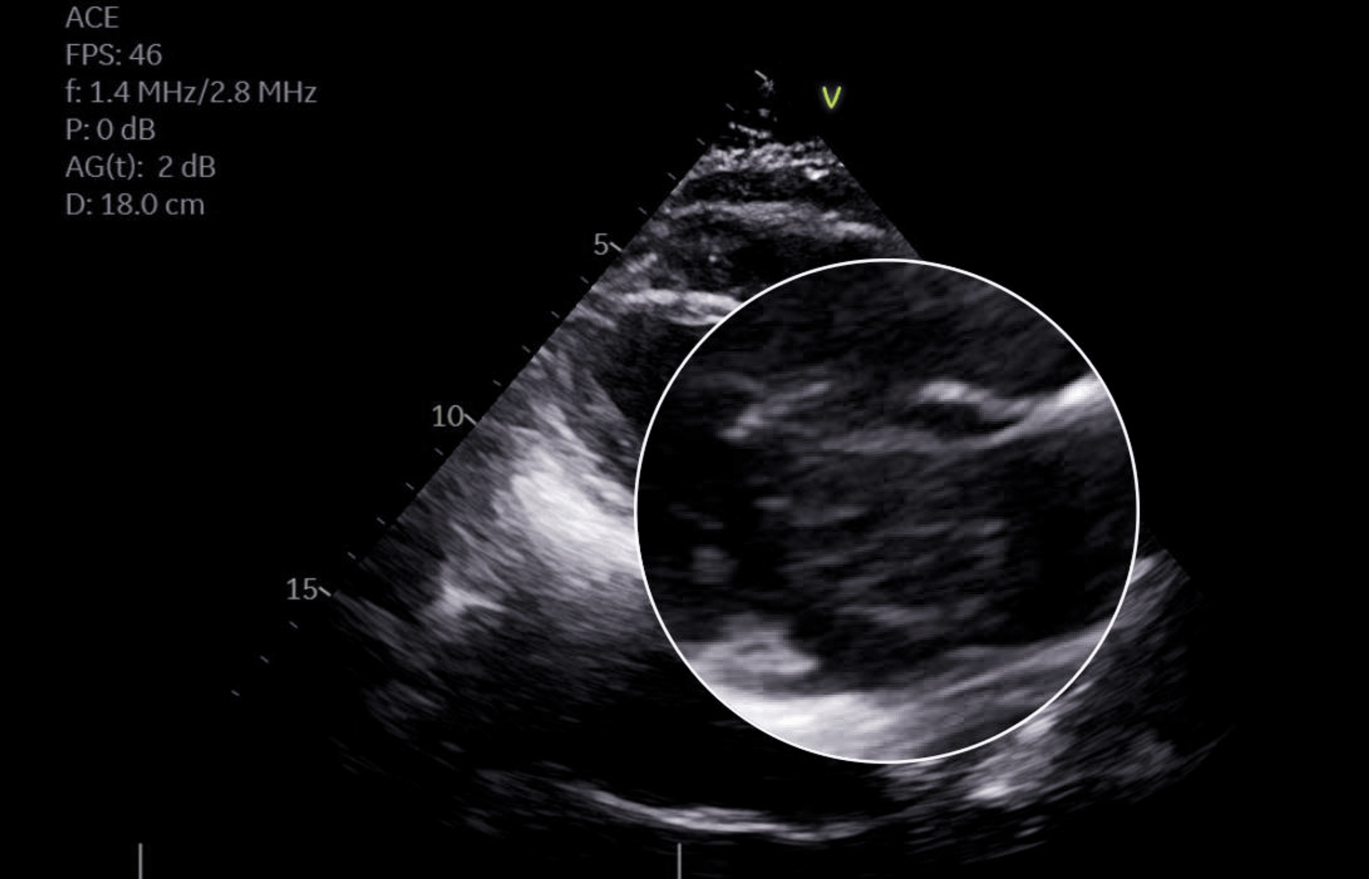
Magnified portion of echocardiogram showing a pedunculated mass in the left atrium

The patient was immediately called to the hospital, where a transesophageal echocardiogram (TTE) showed a mass (4x4 cm) in the left side of the interatrial septum and normal contractility (Figures 2, 3). Cardiothoracic surgery was consulted. The patient underwent resection of the mass (6x6 cm) with pericardial patch reconstruction of the interatrial septum. Repeat TEE demonstrated no residual mass or structural defect. Pathology showed spindled cells distributed in the myxoid matrix, positive for calretinin, consistent with a cardiac myxoma, and not worrisome for malignancy. On the follow-up visit, the patient endorsed improved intermittent DOE and dizziness on severe exertion.

## Discussion

Dyspnea on exertion

DOE serves as a crucial symptom warranting a thorough evaluation, given its association with various medical conditions [8]. This discussion elucidates the respiratory, cardiovascular, and systemic causes of DOE and the intricate mechanisms contributing to this symptom.

Respiratory and Cardiovascular Causes

DOE can arise from a variety of respiratory and cardiovascular conditions. Respiratory causes include COPD, asthma, pneumonia, pulmonary embolism, and lung abnormalities like pneumothorax or cancer [2]. On the cardiovascular side, acute coronary syndrome, heart failure, and arrhythmias are significant contributors to DOE [9,10]. Accurate diagnosis and management depend on recognizing these potential underlying issues, highlighting the necessity of a holistic approach that includes both cardiac and respiratory evaluations for a complete assessment.

Other Systemic Illnesses

Beyond the respiratory and cardiovascular systems, various systemic illnesses contribute to DOE [11]. Metabolic acidosis, anemia, acute renal failure [12], and conditions like thyrotoxicosis [13] or liver cirrhosis [14] can influence overall physiological balance. Recognizing these systemic factors is crucial for a comprehensive evaluation.

Evaluation 

A systematic diagnostic approach is imperative, beginning with a rapid assessment of the patient's airway, breathing, and circulation status and subsequent comprehensive evaluation [4,8]. The initial use of a chest X-ray might allow for the differentiation of cardiac and pulmonary processes, guiding subsequent diagnostic tests such as echocardiography, ECG, and exercise stress testing [11].

Pulmonary Evaluation

Discussing pulmonary evaluation for a patient with mild COPD who was ultimately diagnosed with atrial myxoma underscores the need for meticulous diagnostic efforts. It illuminates the necessity of extensive respiratory testing to exclude usual dyspnea causes before uncovering less evident conditions, such as atrial myxoma. Spirometry serves as a fundamental tool for lung function assessment, while supplementary tests like lung volumes and diffusion capacity offer further pathological insights [15]. Additionally, arterial blood gas analysis is crucial for assessing acid-base balance and detecting issues such as pulmonary embolism, especially when PaO2 is low [2,15]. This comprehensive approach is vital in navigating the complexities of diagnosing dyspnea, ensuring that less common etiologies are considered alongside more prevalent ones.

Specific Conditions and Hypoxia Evaluation

The evaluation of conditions like pulmonary embolism and chronic thromboembolic pulmonary hypertension at times requires specific tests such as D-dimer, leg ultrasound for detecting deep vein thrombosis, V/Q (ventilation/perfusion) scans for assessing lung ventilation and blood flow, and cardiac catheterization to evaluate pulmonary artery pressures and rule out heart-related causes of symptoms [15]. Assessing hypoxia includes analyzing oxygen saturation to identify possible causes, such as carbon monoxide poisoning [15]. The oxygen delivery equation further aids in quantitatively evaluating oxygen transport mechanisms, incorporating factors like hemoglobin level, oxygen saturation, cardiac output, and oxygen partial pressure, offering insights into the physiological underpinnings of dyspnea [16].

Further Assessment and Guiding Principles

In the case of a patient with mild COPD and atrial myxoma presenting with DOE, the utility of a cardiopulmonary exercise test (CPET) highlights the importance of targeted assessment when the cause of dyspnea remains unclear [17]. This approach exemplifies the need for diagnostics tailored to clinical suspicions and patient history, emphasizing a cost-effective and patient-focused strategy [18]. A thorough evaluation, integrating respiratory, cardiovascular, and systemic considerations, is key to accurately diagnosing and managing DOE, ensuring specific conditions like atrial myxoma are adequately addressed.

Chronic obstructive pulmonary disease

In our case, the patient's presentation of DOE initially suggested a primary respiratory issue, potentially exacerbated by their mild COPD. COPD is a multifaceted inflammatory condition that compromises airway and lung function, primarily through mechanisms such as oxidative stress and protease-antiprotease imbalance, which are significantly influenced by environmental irritants like smoking [19]. These processes lead to structural changes, notably emphysema, characterized by the destruction of alveolar structures and a decrease in lung elasticity, facilitating airway collapse during exhalation [20,21].

COPD causes a reduction in forced expiratory volume (FEV1) due to inflammation and airway obstruction [19,20]. Tissue destruction leads to airflow limitation and impaired gas exchange [20]. Imaging studies reveal hyperinflation of the lungs due to air trapping during exhalation, contributing to elevated carbon dioxide (CO2) levels. As the disease progresses, impaired gas exchange can lead to CO2 retention, and pulmonary hypertension may occur due to diffuse vasoconstriction from hypoxemia [20]. Acute exacerbations are common, triggered by factors like bacterial or viral pneumonia or environmental irritants, leading to increased inflammation and air trapping [22]. Treatment during exacerbations often involves corticosteroids and bronchodilators, requiring prompt medical attention [22].

In COPD, exertional dyspnea (difficulty breathing during exercise) results from an imbalance between the increased demand for respiratory effort and the reduced capacity of the respiratory system [19-22]. Physiological ratios, such as ventilation to maximum capacity (VE/MVC) and esophageal pressure (Pes/Pes, max), correlate with dyspnea intensity [1]. Diaphragmatic electromyography (EMGdi/EMGdi, max) reflects increased inspiratory neural drive (IND) in COPD, with a constant dyspnea-EMGdi/EMGdi, max slope across different lung conditions [1,19]. This suggests that dyspnea in COPD is associated with the mismatch between the demand for increased respiratory effort and the decreased capacity of the respiratory system.

Myxoma

Atrial myxomas, the most prevalent primary cardiac tumors, occur infrequently in the general population, with an incidence rate reported to be around 0.5 per 100,000 individuals annually. Despite their rarity, over 75% originate from the left atrium, primarily at the mitral annulus or the fossa ovalis border of the interatrial septum [10]. Right atrium involvement occurs in approximately 20% of cases, while 5% manifest in both atria and the ventricle, potentially causing complications such as obstruction, emboli, and constitutional symptoms like fever and weight loss [10]. A comprehensive approach involving diagnosis through imaging and the effectiveness of surgical intervention in addressing the complications associated with these cardiac tumors are pivotal in the management of atrial myxomas [5-10].

Cardiac myxomas (CMs) are common primary neoplasms in adults, typically presenting as undifferentiated atrial masses attached to the left side of the atrial septum. Despite nonspecific symptoms, a classic triad of constitutional, embolic, and obstructive or cardiac symptoms characterizes CMs [5,6,23]. Diagnostic methods include intentional or incidental discovery through echocardiogram, CT scan, or cardiac MRI [23]. A definitive diagnosis involves macroscopic and histopathological assessment, confirming positivity for endothelial cell markers like CD31 and CD34 [23]. Swift surgical resection is crucial for a positive prognosis [23].

Emerging from the left atrium, cardiac myxomas can affect any cardiac chamber and present as either polypoid or papillary forms, each associated with distinct clinical features [5]. The histogenesis of myxomas is debated, centering around their origin from primitive pluripotent mesenchymal cells, and their clinical manifestation includes intracardiac obstruction, embolic events, and constitutional symptoms mimicking mitral or tricuspid stenosis [5,24].

Echocardiography is the primary diagnostic tool, revealing echogenic polypoid or papillary masses attached to the interatrial septum [24]. Surgical excision remains the cornerstone of treatment, ensuring a favorable prognosis with low operative mortality and recurrence rates [5,23,24]. In the context of diagnosing and treating atrial myxomas, the Carney complex introduces a critical genetic perspective. This syndrome, associated with multiple neoplasms and mutations in the PRKAR1A gene, underscores the importance of considering genetic factors in patients with atrial myxomas [25]. 

Timely and accurate diagnosis is crucial, with echocardiography playing a central role in intentional or incidental detection. Surgical excision is the primary mode of treatment and is essential for a positive prognosis in managing cardiac myxomas.

## Conclusions

The case exemplifies the diagnostic challenges in DOE, particularly when common conditions like COPD coexist with rare etiologies such as atrial myxoma. It illustrates the necessity for a thorough evaluation, advocating for the inclusion of transthoracic echocardiography early in the diagnostic process to uncover less obvious causes. This case further, stresses the importance of considering both respiratory impacts, as detailed in the discussion on COPD, and the nuances of cardiac tumors, like the varied presentations of atrial myxomas. The goal is to encourage healthcare providers to broaden their differential diagnoses, incorporating both common and rare conditions, and to consider comprehensive imaging to facilitate early detection and intervention for unusual pathologies.
